# Wearables for Monitoring and Postural Feedback in the Work Context: A Scoping Review

**DOI:** 10.3390/s24041341

**Published:** 2024-02-19

**Authors:** Vânia Figueira, Sandra Silva, Inês Costa, Bruna Campos, João Salgado, Liliana Pinho, Marta Freitas, Paulo Carvalho, João Marques, Francisco Pinho

**Affiliations:** 1 Escola Superior de Saúde do Vale do Ave, Cooperativa de Ensino Superior Politécnico e Universitário, Rua José António Vidal, 81, 4760-409 Vila Nova de Famalicão, Portugal; sandra.silva@ipsn.cespu.pt (S.S.); a28166@alunos.cespu.pt (I.C.); a28145@alunos.cespu.pt (B.C.); a28906@alunos.cespu.pt (J.S.); liliana.pinho@ipsn.cespu.pt (L.P.); marta.goncalves@ipsn.cespu.pt (M.F.); jsantos.marques@cespu.pt (J.M.); francisco.pinho@ipsn.cespu.pt (F.P.); 2 H2M—Health and Human Movement Unit, Polytechnic University of Health, Cooperativa de Ensino Superior Politécnico e Universitário, CRL 4760-409 Vila Nova de Famalicão, Portugal; 3 Research Centre in Physical Activity, Health and Leisure, Faculty of Sport, University of Porto, Rua Dr. Plácido da Costa, 91, 4200-450 Porto, Portugal; 4 School of Health Sciences, University of Aveiro, 3810-193 Aveiro, Portugal; 5 Department of Medical Sciences, University of Aveiro, 3810-193 Aveiro, Portugal; 6 Center for Rehabilitation Research (Cir), R. Dr. António Bernardino de Almeida 400, 4200-072 Porto, Portugal; 7 Center for Translational Health and Medical Biotechnology Research, School of Health, Polytechnic Institute of Porto, 4200-072 Porto, Portugal; paulocarvalho@ess.ipp.pt

**Keywords:** wearables, feedback, posture, work-related musculoskeletal disorders, workstation

## Abstract

Wearables offer a promising solution for simultaneous posture monitoring and/or corrective feedback. The main objective was to identify, synthesise, and characterise the wearables used in the workplace to monitor and postural feedback to workers. The PRISMA-ScR guidelines were followed. Studies were included between 1 January 2000 and 22 March 2023 in Spanish, French, English, and Portuguese without geographical restriction. The databases selected for the research were PubMed^®^, Web of Science^®^, Scopus^®^, and Google Scholar^®^. Qualitative studies, theses, reviews, and meta-analyses were excluded. Twelve studies were included, involving a total of 304 workers, mostly *health professionals* (n = 8). The remaining studies covered *workers in the industry* (n = 2), in the *construction* (n = 1), and *welders* (n = 1). For assessment purposes, most studies used *one* (n = 5) or *two sensors* (n = 5) characterised as *accelerometers* (n = 7), *sixaxial* (n = 2) or *nonaxial* *inertial measurement units* (n = 3). The most common source of feedback was the *sensor itself* (n = 6) or *smartphones* (n = 4). *Haptic feedback* was the most prevalent (n = 6), followed by *auditory* (n = 5) and *visual* (n = 3). Most studies employed prototype wearables emphasising kinematic variables of human movement. Healthcare professionals were the primary focus of the study along with haptic feedback that proved to be the most common and effective method for correcting posture during work activities.

## 1. Introduction

Currently, work-related musculoskeletal disorders (WRMSDs) are the most prevalent occupational health problem worldwide [1], and they are also the most common in the European Union (EU) [2,3], affecting three out of five workers [4]. Indeed, it is considered a public health problem [5] with multifactorial causes [6,7], resulting from the complex interaction between individual, biomechanical, organisational and psychosocial risk factors [6,8].

WRMSDs affect individuals in all aspects of their lives—as well as companies, society and the economy [2,9,10]—as they result in higher healthcare costs, reduced productivity, increased absenteeism, lower job satisfaction, and reduced physical, psychological, and social well-being of workers [7,11]. In fact, some disorders have a lasting impact on the worker’s life, limiting them in their daily activities and preventing their return to work due to permanent disability caused by pain and decreased functionality [5].

Recently, an increase in WRMSDs has been observed due mainly to mechanical overload [1]. Prolonged inappropriate working postures, tasks with high physical demands, repetitive and meticulous work gestures, and an intense work pace with few breaks have been reported as the main causes of WRMSDs [3,8,9]. Additionally, the demographic change in the workforce [12] has led to an increase in the number of older workers, which may have also contributed to the increase in WRMSDs [1,2].

Given the musculoskeletal system overload [1], real-time monitoring and postural correction in the workplace [13] are urgent. This will minimise the harmful effects of some postures, resulting from the misalignment of body segments with the line of gravity [14]. Posture is a highly complex, variable, and dynamic system that can respond to minimal psychophysical and socioenvironmental perturbations [15]. Due to these features, wearable technology has emerged as a viable alternative with high potential for real-world context implementation [16,17,18]. The projection of wearables, also referred to in the literature as wearable sensors and defined as electronic devices integrated into clothing and/or other accessories that comfortably adapt to the human body [19] would seem to be a valid proposal for improving working conditions, early identification of WRMSDs risks, increased work efficiency, and promoting well-being [20].

Inertial measurement unit (IMU) is the common underlying technology for most wearables [7,21,22]. It is possible to identify static and dynamic improper postures that are maintained for long periods [23,24] through discrete and continuous monitoring of body posture in real-world settings [21,25] in a shorter timeframe/in a shorter time [5]. Other advantages of wearables that make them robust for integration in monitoring work activity include objective, reliable, and accurate results [26] which provide a trustworthy and realistic assessment of work-related conditions [27]; low cost; lightweight design; small size; portability; and energy efficiency [28].

Furthermore, some wearables have the feature to provide real-time corrective sensory feedback when adopting inadequate postures [3]. This feedback can be auditory (typically conveyed through diverse auditory channels), visual (usually displayed by screens or projectors), haptic (application of vibratory stimulus), or a combination of these, providing information based on performance or outcome [29,30]. Haptic feedback stands out as the most common choice [9,31,32] and also as the most advantageous option given its discreet nature [29]. It contrasts with auditory and visual feedback, which can be perceived by other workers, affecting their concentration [9]. Regarding the feedback signal, it can be provided at the end of the task, referred to as terminal feedback, or in real-time, known as concurrent feedback [29]. The latter has the advantage of promoting immediate changes in work postures [33]. However, regardless of the type of corrective feedback, it promotes greater postural self-awareness, allowing for the minimisation of inadequate postures and, consequently, reducing the musculoskeletal overload [13]. This factor emphasises the potential for the urgent implementation of wearables in different work contexts to reduce the incidence of WRMSDs and the associated healthcare costs [9,32]. Nevertheless, still in their early stages, wearables present disadvantages and/or challenges such as accuracy, technical functionality, and usability [30]. Factors such as battery life, long-term comfort, preparation time (donning and doffing; changing or recharging batteries) and the stability of wireless communication contribute to workers’ reluctance to use such devices [2,30,34,35]. Recent studies have tried to address the challenges of wearables by improving the usability and effective monitorization of human movement, as well as the autonomy of wearables, which are crucial for practicality and commercial viability, with the aim of establishing wearables as common tools in workplace settings [36]. The potential of wearables in preventing and minimising WRMSDs is supported by growing scientific evidence on postural monitoring wearables in various contexts [20,36,37,38,39]. Despite advances, challenges remain in wearables for simultaneous postural monitoring and feedback in real-world scenarios, emphasising the need for continued research. Furthermore, the importance of wearables in minimising WRMSDs positions them as an emerging field of research. It is important to understand and summarise all the current evidence to contribute to the development of new methods to promote the worker’s health and quality of life.

Therefore, the objectives of this study were established to comprehensively identify, synthesise, and characterise the wearables used in the workplace to monitor and provide postural feedback to workers. Specifically, the study aimed to identify and summarise the variables used to detect postural changes, the location of the sensors, the attachment methods, and the number of sensors employed. It also aimed to analyse the type and source of corrective feedback used, the professions/occupations, and the contexts in which these wearables have been applied to contribute to the reduction of WRMSDs.

Review questions

The main review question of this study was: “What wearables have been used for postural monitoring and correction in the workplace?”

The review sub-questions are listed as follows:

- What variables have been considered in the identification of postural changes?
- What location, type of fixation, and number of sensors have been used to monitor and provide postural feedback in the work context?
- Which occupations or work tasks have been analysed using wearables to monitor and provide postural feedback in the work context?
- Among the identified wearables, what type and source of feedback is being used for postural correction?
- What results have been reported following the application of postural feedback in a work context?

## 2. Materials and Methods

In this scoping review, the Preferred Reporting Items for Systematic Reviews and Meta-Analyses extension for Scoping Reviews (PRISMA-ScR) guidelines [40] and the methodology proposed by the Joanna Briggs Institute (JBI) manual for evidence synthesis were followed [41]. The protocol was registered on the Open Science Framework, which the review questions and the methodology were specified, Supplementary Materials.

### 2.1. Eligibility Criteria

The Population, Concept, Context (PCC) strategy JBI [41], as reported in Table 1, was used to develop the search strategy and to the define the eligibility criteria for study inclusion (Table 1).

The potentially eligible studies were identified in the databases on 22 March 2023, without geographical restrictions, in Spanish, French, English, and Portuguese. Unpublished grey literature was also searched.

Studies in humans published between 1 January 2000 and 22 March 2023 were included, while qualitative studies, literature reviews, systematic reviews, meta-analyses, and theses were excluded.

### 2.2. Information Source

Three databases, PubMed^®^, Web of Science™ (WOS), Scopus^®^ (non-grey literature) and a scholarly literature web search engine (Google Scholar^®^) were used. A manual search was also conducted based on the relevant bibliography consulted to identify other eligible studies. The strategies used in the different databases are described in Table 2. Due to the specificities and their filters, the term “humans” and the language were specified in advance for PubMed^®^.

### 2.3. Selection of Evidence Sources

Each of the three investigators (JS, IC, BC) performed the search simultaneously in the same databases, following the defined strategies. No discrepancies were found during the data extraction process, which was imported into Mendeley^®^ software, version 1.19.8, (Elsevier), and duplicates were removed. After this step, to facilitate the screening process and to confirm the presence of any duplicates not identified by the software, all extracted articles were imported into Microsoft^®^ Excel^®,^ version 2304. All reviewers conducted a pilot study until a minimum consensus of 75% was reached in the selection of 25 units of analysis for title and abstract screening, based on a priori eligibility criteria. After concluding this process, the screening by title and abstract was performed, identifying the studies as “included” or “excluded”.

**Figure 1:**
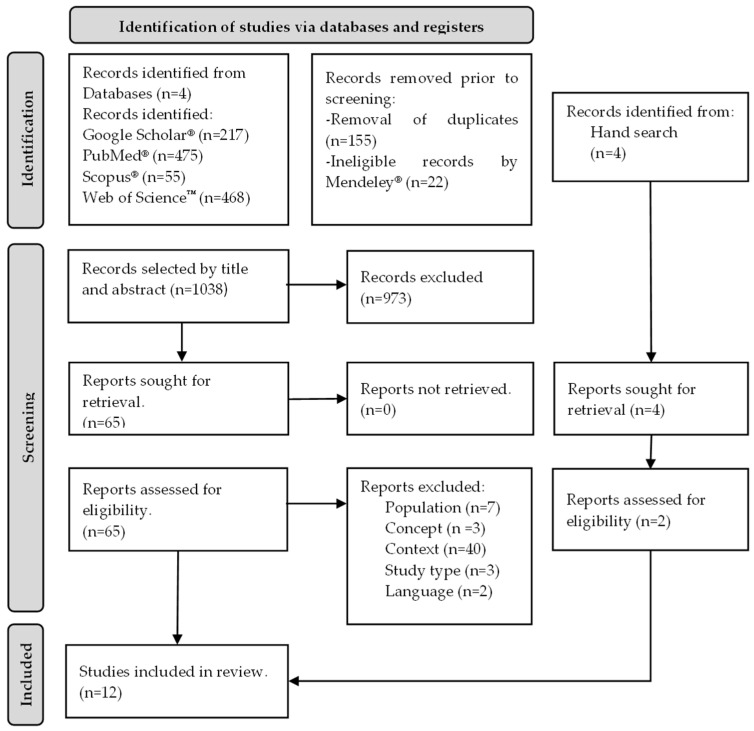
Flowchart of included studies, adapted from PRISMA ScR statement [42].

The full text of the included articles was read to exclude those that did not meet the eligibility criteria. In case of disagreement, a fourth reviewer (VF) was consulted. This process is detailed in the form of a PRISMA-ScR flowchart (Figure 1).

### 2.4. Data Extraction

Data were extracted for inclusion in the evidence table with the general characteristics of the eligible studies (Table 3): authors/year, study design, sample characterization, context/setting, occupation/work task, and region and anatomical plane under analysis. Additional data were also extracted to provide a better characterisation of the wearables considered in the included studies, which are explained in Table 4: type, number of sensors (wearables) and signal or variable under study, sensor placement and fixation, and feedback source and type. The results after feedback application are in Table 5.

### 2.5. Data Presentation

A narrative report and a tabular form were produced to summarise the data around the main research question.

## 3. Results

A total of 1215 units of analysis were identified, out of which 22 were deemed ineligible by the automated tools during the transfer to Mendeley^®^. Of these, 155 duplicate units were removed. After title and abstract screening, 973 articles were excluded because they did not meet the predefined eligibility criteria. As a result, 65 articles were read in full, and 55 of these were excluded. The reasons for study exclusion were as follows: population (n = 7), concept (n = 3), context (n = 40), study type (n = 3), and language (n = 2). The remaining 10 studies were included with 2 additional manual searches, resulting in a total of 12 studies (Figure 1).

A total of 304 workers were included in this scoping review [43,44,45,46,47,48,49,50,51,52,53,54] (Table 3). Eight of the 12 studies included participants of both sexes [43,44,45,46,50,52,53,54], two studies included only female participants [49,51], and the other two did not specify the participants’ sex [47,48] (Table 3).

**Table 3:** Study design, characteristics of the participants, setting, occupation/work task, and region and anatomical plane under analysis.

Author, Year	Study Design	Sample	Context/ Setting	Occupation/ Work Task	Region and Anatomical Plane Under Analysis
Ribeiro et al., 2014 [43]	Randomized controlled trial	n=62 (F=57 e M=5) $\bar{x}$ = 49.6 ± 12.4 years Eligibility: with and/or without low back pain	Healthcare institution	Healthcare and administrative professionals	Lumbopelvic region; Sagittal and frontal plane
Thanathornwong et al., 2014 [44]	2 × 2 crossover randomized trial	n=16 (F=14 e M=2) $\bar{x}$ = N/S (Min: 25 and Max: 30 years) Eligibility: dentists working at least 6 h daily.	Hospital	Dentists during molar surgery	Cervical and upper trunk; Sagittal and frontal plane
Thanathornwong et al., 2014 [45]	2 × 2 crossover randomized trial	n=16 (F=14 e M=2) $\bar{x}$ = N/S (Min: 21 and Max: 23 years) Eligibility: minimum practice of 6 h daily as a dentist and scoring over 70% on the applied questionnaire.	Real N/S	Dental students	Cervical and upper trunk; Sagittal and frontal plane
Thanathornwong and Suebnukarn, 2015 [46]	2 × 2 crossover randomized trial	n=16 (F=8 e M=8) $\bar{x}$ = N/S (Min: 21 and Max: 23 years) Eligibility: minimum practice of 6 h daily in dental work tasks.	Dental clinic	Dental students	Upper trunk; Sagittal and frontal planes
Zhao et al., 2015 [47]	Observational descriptive study	Healthcare caregivers n=N/S $\bar{x}$ = N/S	Real N/S	Healthcare caregivers	Spine (++ lumbar); Sagittal plane.
Yan et al., 2017 [48]	Validation study	n=N/S $\bar{x}$ = N/S	Laboratory context and real construction context	Construction workers during brick lifting and steel rod handling tasks	Head, Cervical, and thoracic region; Sagittal plane.
Doss et al., 2018 [49]	Analytical cross-sectional observational study	n=10 (F=10) $\bar{x}$ = 26.1 ± 9.1 years Eligibility: nursing students without a history of back pain.	Clinical experience in a N/S context	Nursing students/patient transfer	Trunk; Sagittal plane.
Lins et al., 2018 [50]	Pilot study (experimental)	n = 11 (M = 8 e F = 3) $\bar{x}$ = N/S Eligibility: N/S	Real N/S	Welders	Cervical, thoracic, lumbar, scapular waist, elbows, wrists, and knees regions. N/S Plane
Bootsman et al., 2019 [51]	Analytical cross-sectional observational study	n=13 (F=13) $\bar{x}$ = 39.77 ± 13.6 years Eligibility: healthy nurses (without low back pain) who do not engage in sedentary work tasks.	Hospital	Nurses (9 nurses from the Neonatal Intensive Care Unit and 4 home care nurses)	Lumbar spine; Sagittal plane.
Lind et al., 2020 [52]	Observational study	n=15 (F=3 e M=12) $\bar{x}$ = 23.33 ± 2.9 years Eligibility: workers without discomfort and/or work-related musculoskeletal injuries that could hinder order-picking tasks.	Multinational vehicle construction company	2 employees in logistics applications and 13 employees in order picking and assembly tasks	Dominant upper limb and trunk; Sagittal plane.
Ribeiro et al., 2020 [53]	Randomized controlled trial	n=130 (F=84.6 M=15.4) $\bar{x}$ = 45.3 ± 13.2 years Eligibility: adult healthcare professionals, with or without current presence (or history) of low back pain, currently performing their work tasks normally.	Continuing care institutions and hospitals	Healthcare professionals	Lumbopelvic region; Sagittal and frontal planes.
Lind et al., 2023 [54]	Analytical observational study	n=15 (M=14 e F=1) $\bar{x}$ = 30.8 ± 11.5 years Eligibility: healthy workers without a history of pain that would hinder their W.T	Warehouse	Warehouse workers	Trunk; Sagittal plane.

n—sample; F—female; M—male; $\bar{x}$—mean age; Min—minimum; Max—maximum; W.T.—work tasks; N/S—nt specified.

### 3.1. Context/Setting, Occupation/Work Task and Region and Anatomical Plane under Analysis

**Figure 2:**
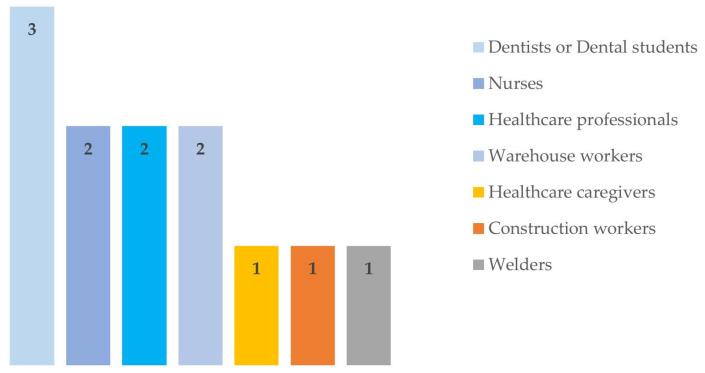
Characterisation of the context/work task.

With regard to occupation, most of the tasks involved healthcare professionals, namely nurses [49,51], dentists or dental students [44,45,46], healthcare caregivers [47], and unspecified healthcare professionals [43,53]. Additionally, the tasks of welders [50], construction workers [48] and warehouse workers [52,54] were also assessed (Table 3) (Figure 2).

**Figure 3:**
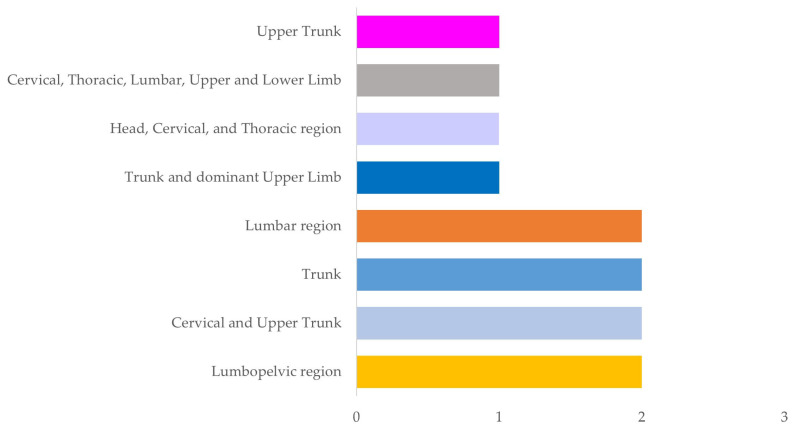
Anatomical region analysed.

The main area for assessment was the trunk, with the thoracic region being the most favoured [44,45,46,48,54]. This was assessed in isolation [46,49,54] or in combination with different regions of the trunk [44,45,48] or upper limb [52]. On the other hand, other studies focused more on the analysis of the lumbar [47,51] or lumbopelvic region). It should be noted that only the study by Lins et al. [50] assessed all regions of the trunk, upper limbs (elbow and wrist), and lower limbs (knee) (Table 3) (Figure 3). Except Lins et al. [34], who did not specify the level of analysis, all studies assessed movement in the sagittal plane, and in some cases, this analysis was combined with the assessment of movement in the frontal plane [43,44,45,46,53].

### 3.2. Type, Number of Sensors (Wearables), and Signal or Variable under Study

**Figure 4:**
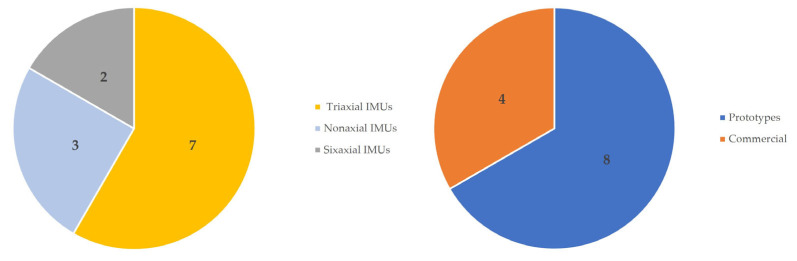
Type of sensors.

The majority of the studies used IMUs with accelerometers only [43,44,45,46,47,49,53], and mostly sensors in the form of prototypes (n = 8) [44,45,46,49,50,51,52,54] (Figure 4). Relating to the number of sensors, 5 studies used 1 sensor [43,46,47,53], 5 other studies used 2 sensors [44,45,48,49,51,54], 1 study used 3 sensors [52] and another study used 15 sensors [50]. These were placed along various body segments, specifically the cervical, thoracic, lumbar, scapular-waist, elbows, wrists, and knees regions (Table 4).

**Table 4:** Characterization of posture analysis and feedback wearables from the included studies.

Authors (Year)	Type, Number of Sensors (Wearables), and Signal or Variable under Study	Sensor Placement and Fixation	Feedback Source and Type
Ribeiro et al., 2014 [43]	Triaxial IMU (Accelerometer) 1 sensor (Commercial) Acceleration and linear position.	Laterally fixed on the participant’s belt at the level of the lumbopelvic region.	Integrated feedback in the sensor itself. Simultaneous intermittent auditory feedback.
Thanathornwong et al., 2014 [44]	Triaxial IMU (Accelerometer) 2 sensors (Prototype) Linear position.	Fixed on the PPE (visor) and posteriorly fixed on the worker’s uniform at the thoracic region (T4).	N/S Feedback. Visual feedback at the end.
Thanathornwong et al., 2014 [45]	Triaxial IMU (Accelerometer) 2 sensors (Prototype) Linear position.	Fixed on the PPE (visor) and posteriorly fixed on the worker’s uniform at the thoracic region.	N/S Feedback. Visual feedback at the end.
Thanathornwong and Suebnukarn, 2015 [46]	Triaxial IMU (Accelerometer) 1 sensor (Prototype) Trunk flexion, extension, and inclination linear position.	Fixed on the worker’s uniform, posteriorly at the thoracic region.	Integrated feedback in the sensor itself. Haptic feedback.
Zhao et al., 2015 [47]	Triaxial IMU (Accelerometer) 1 sensor (Commercial) Acceleration and linear position.	Smartwatch worn on the wrist (side determined by the participant).	Integrated feedback in the sensor itself. Haptic feedback.
Yan et al., 2017 [48]	Nonaxial IMU (triaxial Accelerometer, triaxial Gyroscope, and triaxial Magnetometer) 2 sensors (Commercial) Angular and linear acceleration.	Posteriorly fixed on the protective helmet and safety harness and vest at the thoracic region (between T1 and T2).	Smartphone-based feedback. Auditory feedback.
Doss et al., 2018 [49]	Triaxial IMU (Accelerometer) 2 sensors (Prototype) Trunk acceleration and linear position.	Tape-fixed at the thoracic (vest) and lumbar (belt) regions.	Smartphone-based feedback. Auditory feedback.
Lins et al., 2018 [50]	Sixaxial IMU (triaxial Accelerometer and triaxial Gyroscope) 15 sensors (Prototype) Linear and angular acceleration.	Tape-fixed on the worker’s uniform, specifically at the cervical, thoracic, lumbar, scapular waist, elbows, wrists, and knees regions.	Integrated feedback in the sensor itself. Haptic feedback.
Bootsman et al., 2019 [51]	Nonaxial IMU (triaxial Accelerometer, triaxial Gyroscope, and triaxial Magnetometer) 2 sensors (Prototype) Displacement, angular velocity, and linear acceleration.	Posteriorly fixed on the work uniform (in a built-in pocket) at the lumbar region, specifically between L1 and L5.	Smartphone-based feedback. Visual, auditory, and haptic feedback.
Lind et al., 2020 [52]	Nonaxial IMU (triaxial Accelerometer, triaxial Gyroscope, and triaxial Magnetometer) 3 sensors (Prototype) Angular and linear displacement of the trunk and dominant upper limb.	The IMU is bilaterally fixed on the worker’s arm (built-in pockets) at the deltoid muscle insertion. The haptic sensors are anteriorly fixed through a belt at the thoracic region (between T1–T2) and on the arm through an armband.	Smartphone-based feedback via Bluetooth. Haptic feedback.
Ribeiro et al., 2020 [53]	Triaxial IMU (Accelerometer) 1 sensor (Commercial). Acceleration and linear position.	Fixed on the belt at the lumbopelvic region.	Integrated feedback in the sensor itself. Auditory feedback.
Lind et al., 2023 [54]	Sixaxial IMU (triaxial Accelerometer and triaxial Gyroscope). 1 sensor (Prototype) Acceleration and linear position.	The IMU is fixed in a built-in pocket at the thoracic region (between T1 and T2). The haptic sensor is embedded in a pocket on the T-shirt at the sternum level.	Integrated feedback in the sensor itself. Haptic feedback.

PPE—personal protective equipment; N/S—not specified; IMU—inertial measurement unit.

### 3.3. Sensor Placement and Fixation

**Figure 5:**
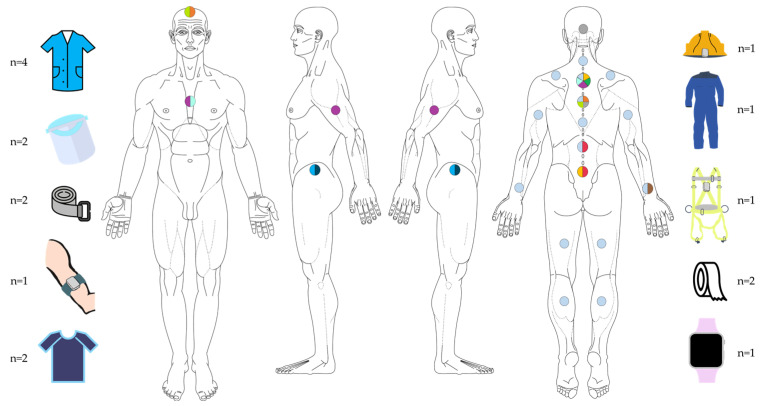
Sensor placement and fixation.

These sensors were attached to accessories or personal protective equipment (PPE), including vests, helmet, thoracic and lumbopelvic belts, uniforms (e.g., pockets) and face protectors (visors) [43,44,45,46,48,49,51,53,54]. In the upper limb (n = 3), sensors were placed on armbands and smartwatches [47,50,52] (Figure 5).

### 3.4. Feedback Source and Type

Regarding the source of feedback, the most common was the sensor itself (n = 6) [43,46,47,50,53,54], followed by smartphones (n = 4) [48,49,51,52]. In addition to the sensor itself, Lins et al. [50] also used a decision support system. Two studies did not specify the source of feedback [44,45].

**Figure 6:**
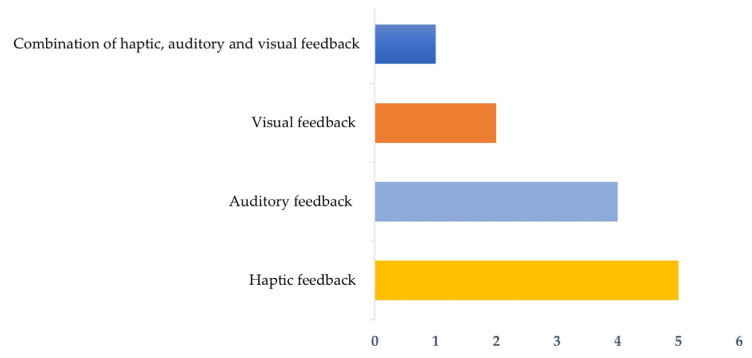
Type of feedback.

The type of haptic feedback was the most identified [46,47,50,52,54], and it is highlighted that the study by Bootsman et al. [51] was the only one that combined all three types of feedback (Figure 6).

### 3.5. Results after Feedback Application

Following feedback application, all included studies showed improvements. A better lumbar posture was demonstrated in two studies [43,51], and reductions in trunk flexion and inclination during work tasks were observed in seven studies [44,45,46,48,49,52,54]. It should be noted that according to Bootsman et al. [51], there were no significant differences between the three types of feedback (Table 5).

**Table 5:** Results of the included studies.

Authors (Year)	Results after Feedback Application
Ribeiro et al., 2014 [43]	In the constant feedback group, there was a reduction in lumbar flexion compared to the control and intermittent feedback groups, with constant feedback being more effective.
Thanathornwong et al., 2014 [44]	The group that received feedback significantly decreased cervical and upper thoracic extension, as well as reduced the likelihood of WRMSDs in the post-test.
Thanathornwong et al., 2014 [45]	There were statistically significant differences in reducing cervical extension with posterior alignment.
Thanathornwong and Suebnukarn, 2015 [46]	There was a decrease in trunk flexion and inclination in the upper body in the feedback group compared to the group without feedback.
Zhao et al., 2015 [47]	The system developed by the authors can be used to improve safe patient handling with the use of discrete tactile feedback in real time.
Yan et al., 2017 [48]	After an adaptation period of nearly a day to the proposed PPE, there was an improvement in tasks, indicating the effectiveness of self-awareness and self-regulation strategy.
Doss et al., 2018 [49]	After using feedback, statistically significant differences were observed in the task of transferring from bed to chair, including a decrease in the average time to complete the task, a reduction in peak trunk flexion and rotation, and triaxial speed and acceleration.
Lins et al., 2018 [50]	The results indicate that the ideal pulse length for haptic feedback application is about 150 ms, repeated 2 or 3 times within the sequence for maximum attention.
Bootsman et al., 2019 [51]	Improvement in lumbar posture compared to the group that did not receive any type of feedback, with no significant differences between the different types of feedback.
Lind et al., 2020 [52]	Decrease in elevation of the dominant upper limb and trunk flexion immediately after haptic feedback, which was maintained after its removal.
Ribeiro et al., 2020 [53]	There were no statistically significant differences between the groups with the application of auditory feedback to limit the threshold of trunk flexion.
Lind et al., 2023 [54]	Decrease in flexion and inclination of the upper trunk in the group with feedback compared to the group that did not receive haptic feedback.

WRMSDs—work-related musculoskeletal disorders**;** PPE—personal protective equipment; ms—milliseconds.

## 4. Discussion

This scoping review aimed to identify, synthesise, and characterise the wearables used in the workplace to monitor and provide postural feedback in workers. Indeed, taking into consideration the global increase in the prevalence of WRMSDs with an ageing workforce [10], it seems justified to conduct studies in work environments that incorporate devices aimed to reduce the effects of the work tasks’ physical demands and alerting workers to adopt correct postures [5,55].

### 4.1. Wearables and Variables of Human Movement in the Workplace Settings

This review exclusively considered the integration of studies in real-world contexts involving work tasks, whereas most wearable studies are typically conducted in laboratory settings [2]. Certainly, it is important to highlight that the monitorisation and correction of posture in real environments, which is currently feasible, is only possible due to the exponential development of technology that allows the use of minimalist portable sensors (in particular inertial sensors) which are commonly used in biomechanical studies and integrated into wearables devices [28]. The gold standard for kinematic assessment, particularly joint range assessment, which includes digital goniometers that are easy to access and use, as well as optical motion capture systems, have some disadvantages when it comes to being integrated into a workplace context. As far as goniometers are concerned, they are usually used more for kinematic measurements of the wrist, especially twin-axis electrical goniometers placed on the targeted body segment [54]. Their accuracy depends on the expertise of the user, while optical capture systems are more limited to laboratory settings [56]. Therefore, the use of IMUs seems to be more consistent with greater potential for application in a workplace context, providing more reliable and trustworthy results [2]. It is widely recognised that real tasks, as opposed to simulated tasks, appear to involve a greater variability and complexity of movement that is difficult to replicate in controlled environments [57]. In this sense, wearables have emerged as a viable solution to address these gaps due to their ability to provide objective measurements of human movement, particularly posture [22].

Furthermore, Buisseret et al. [25] stated that wearables add significant value to the analysis of human movement in real and dynamic situations, which justifies their application in posture monitoring and correction. Paloschi et al. [21] argued that wearables are valid for quantitative measurement of daily occupational posture, with inertial measurement units (9 axis IMUs) being a robust instrument commonly used for this purpose. This statement is supported by Cerqueira et al. [5], who noted that IMUs are an increasingly valid and recognised option for wearable integration due to their many advantages, including three-dimensional motion capture, size and weight, and portability. Choi et al. [58] also argue that IMUs are important tools for managing the health and safety of workers, allowing continuous monitorisation and identification of incorrect postures, with feedback provided when a risky posture is detected. Based on this evidence, it seems reasonable to integrate IMUs into posture monitoring and feedback wearables in the workplace context. It is worth noting that IMUs, which allow for a more comprehensive assessment [7], are the most used type of sensor according to Ciccarelli et al. [2]; Conforti et al. [14]; Donisi et al. [59], and Patel et al. [18]. In contrast, this scoping review found that IMUs with accelerometers only (three-axis IMU-accelerometer) were the most frequently used resource for analysis and corrective feedback in the workplace. These findings are supported by the study by Lee et al. [7] and contradicted by the study by Wang et al. [32], which found an equal use of both types of sensors (three vs. nine axis IMUs). However, these authors emphasised that the choice between different IMUs will always depend on the objective of the study considering the complexity of the assessed task. Therefore, considering that the objective of the different studies included in this scoping review was to identify postural changes based on the defined assumptions, these choices seem to have been appropriate. Using different IMUs, whether triaxial or nonaxial, it is possible to identify body position with different degrees of accuracy. The triaxial IMUs offer a simpler approach and the nonaxial a more complex analysis.

It is widely known that these tools allow the analysis of kinematic parameters of human movement [27,60]. In this study, only kinematic variables—specifically displacement and angular velocity, linear and angular acceleration, and linear position—were analysed individually or in a combined and complementary manner. The analysis of these parameters, although valid and appropriate, could be even more robust and complex if it were possible to associate simultaneous analysis of kinetics. Based on the assumption that muscle fatigue is one of the risks associated with WRMSDs, it is known that Median Frequency (MDF) and Root Mean Square (RMS) analysis in electromyography (EMG) makes it possible to characterise the spectral distribution of power and measure amplitude, respectively, characterising muscle condition, namely fatigue [61]. Thus, integrating EMG on top of triaxial or sixaxial IMUs would provide a more comprehensive analysis and enable early detection and correction of muscle fatigue, minimising the risk of WRMSDs [62]. Studies involving portable EMG sensors in the workplace context have already been developed, tested, and evaluated, suggesting that monitoring human movement in real contexts will soon become increasingly simpler and robust [63,64]. In the absence of this combination, nonaxial IMUs are the most commonly used, as they integrate a triaxial accelerometer, triaxial magnetometer, and a triaxial gyroscope, providing greater robustness and reliability in the analysis of human movement kinematics [2,24]. This type of IMU was used in three studies included in this scoping review, while the other studies relied on six axial or triaxial IMUs. These choices also appear to be valid, as bi-, tri- and six-axial IMUs all have high validities in measuring joint position—particularly flexion/extension movement—and are viable alternatives (probably the only alternative) to the gold standard in occupational contexts [65]. Given that most studies only assessed movement in the sagittal plane, both bi- and triaxial IMUs are appropriate and valid choices. Even those that used triaxial IMUs to analyse joint position in the frontal plane, in addition to the sagittal plane, seem to be a possible choice, although less robust.

However, this simplified information could be more accurate and robust if complemented by the use of a triaxial gyroscope and magnetometer [21,66]. This raises the question of whether the results of the studies by Ribeiro et al. [43,53], Thanathornwong et al. [45], Thanathornwong et al. [44], Thanathornwong and Suebnukarn [46], Zhao et al. [47], and Doss et al. [49] could have been more complete or led to different conclusions due to the instrument used.

### 4.2. Analysis and Feedback in the Workplace: Type, Location, Attachment, and Quantity of Sensors

Photogrammetric methods, which are the gold standard for posture analysis, consist of approaches that are more geared towards laboratory use and are not suitable for monitoring and correcting workers’ postures in their daily lives [22]. On the other hand, indirect methods such as questionnaires or observational methods like REBA or RULA are, according to the available literature, the most used in the workplace context to assess postures and related factors [63,67]. Consequently, the need to develop alternative methods for real-time objective assessment of work tasks and the subsequent adoption of appropriate postures was created [67]. However, based on existing evidence, it is known that workers are often unaware of their posture and frequently adopt incorrect behaviours due to time constraints, task demands, and the need to meet productivity goals [22]. Truly, there is a lack of consensus on correct posture [13], which is generally described as considering the alignment of different body segments about the line of gravity with minimal energy expenditure [13,68]. However, in this scoping review, all included studies started from the assumption that the neutral position, with the head and trunk aligned at 0° relative to the rest of the body, would be considered correct posture. To measure this posture, all studies relied on wearable devices, with heterogeneity in the attachment methods, number and location of sensors used for that purpose.

Most sensors used in the included studies were at the prototype stage despite the growing trend towards commercialisation of wearable devices [22]. In fact, although there is already a wide range of commercially available wearable devices today, most are aimed at physical activity monitoring, posture and physiological parameters, in contrast to the apparent lack of commercial wearable systems that combine postural monitoring and feedback [22]. This seems to be a gap for their integration in the workplace context [2]. This may be due to difficulties not only in accessing such devices but also to the fact that, in most cases, they do not meet usability parameters, which can consequently hinder the acceptance of these instruments by workers and companies, and actually provide data incongruent with reality [2,34]. Furthermore, Jacobs et al. [69] argued that the workers’ acceptance of wearables may be influenced by factors related to the organizational environment as well as individual characteristics and beliefs, although these factors were not mentioned in any of the studies included in this review. Recent studies emphasise the careful design of wearables, using flexible materials such as polybutylene terephthalate and polydimethylsiloxane to provide flexibility, durability and comfort, which provides greater resistance to repetitive movements and could therefore ensure greater practical acceptance of the sensors by workers [36]. Continued research to develop more wearable, practical, portable and autonomous sensors is essential to make their commercialisation feasible and expand their application in the workplace context, helping to minimise WRMSDs) [20,36].

According to Donisi et al. [59], it is observed that the most common and frequent approach to monitor postural changes is the placement of sensors throughout the body. However, this contradicts the findings of this scoping review, where the authors predominantly placed the sensors on the upper trunk, cervical and thoracic spine, and upper limbs, alongside some studies that focused on the lumbar region or the entire body. In truth, these regions have been the target for sensor placement, possibly due to the increased incidence of WRMSDs in this region [70,71]. These results are consistent with the study by Lorenzini et al. [72], which states that the upper limbs and the spine are the most frequently affected body regions, supporting the placement of sensors in these specific regions.

Despite the device placement, also the usability, the comfort and accuracy are key factors in worker acceptance, and current evidence reinforces its importance [5,7,73,74]. Indeed, the sensors attachment is a fundamental role in its usability. Therefore, to meet this requirement, the sensor attachment must fulfil three essential conditions, namely being imperceptible to the worker; having intuitive use; and providing quick, reliable, and easily interpretable information [21]. These criteria are crucial to ensure the effectiveness of wearables, alongside worker acceptance, and can be integrated into accessories or clothing [25]. This approach, which is currently the greatest consensus [75] regarding sensor integration, was found in the studies of Bootsman et al. [51], Lind et al. [52,54], Lins et al. [50], Ribeiro et al. [43,53], and Thanathornwong and Suebnukarn [46].

Other sensor attachment methods can include direct placement on the skin using electrodes that incorporate EMG [76], adhesive tape or elastic straps [33,67], integration into smartwatches, or even integration into personal protective equipment (PPE) such as helmets and visors [60,77,78]. The latter form of attachment is consistent with Yang et al.’s [65] study on construction workers, who used sensors attached to PPE, specifically helmets and safety harnesses or vests. In fact, Choi et al. [58] argue that this is the most suitable location for sensor placement in this occupational activity, as it does not appear to interfere with the work, which can increase usability.

On the other hand, two studies combined sensor placement on the uniform and visor, while one study opted for sensor attachment with adhesive tape on clothing and belt. This heterogeneity in sensor attachment was also confirmed in the study by Lee et al. [7], highlighting the lack of consensus on sensor placement [30]. Indeed, most of the studies included in this scoping review only superficially described the parameters of location and attachment, raising the question of whether this heterogeneity and discrepancy between methods could bias the results obtained. This leaves an open question remaining for future studies: Is there a need to develop a universal protocol for sensor application in common anatomical regions—considering the imprecision in location description, diversity of attachment methods, and material types—to allow for greater robustness in comparability between studies?

One problem that arises when implementing the wearable devices in real-world settings is the resistance of workers and companies to their use. This resistance may be due not only to the inherent slowness of their application but also to the large number of sensors that some of these systems require [30]. This may be a reason why most of the studies included in this scoping focused on the assessment of a single region, with a particular emphasis on the spine, predominantly using one or two sensors, which is consistent with the literature [38,39].

### 4.3. Occupation and Work Tasks

Healthcare professionals such as nurses, dentists, healthcare assistants, and unspecified healthcare professionals were the most studied populations on posture monitorisation and feedback using wearables. The inclusion of these professions in the studies included in this review seems to reveal the existing evidence of high prevalence of musculoskeletal disorders related to occupational activities in the healthcare settings, indicating the need to intervene in this area to minimise their occurrence [70]. Indeed, healthcare professions typically involve demanding work schedules and precise, meticulous, and repetitive tasks, often requiring prolonged static postures that contribute to neuro-musculoskeletal strain and fatigue, particularly among dentists [79,80,81]. This fact is also supported by studies that have found a high prevalence of musculoskeletal disorders in this group [70,82,83], particularly among those with more than 10 years’ clinical practice and working more than 40 h per week [83]. Jacquier-Bret and Gorce [70] also found that dentists are among the professionals most exposed to musculoskeletal disorders, with a higher prevalence of symptoms in the spine, particularly in the cervical and lumbar regions, which is also supported by Blume et al. [82]. In addition, dental students are also susceptible to develop musculoskeletal disorders, and there are studies that support this evidence by linking this susceptibility to the adoption of more sedentary behaviours due to the use of new technologies that decrease their mobility [83,84]. On the other hand, Blume et al. [82] describe an association between musculoskeletal disorders and poor posture adopted by these students during dental procedures, particularly when these involve static postures, especially in the cervical region, due to the limited visibility of the patient’s mouth, which leads to the maintenance of an extended and protracted cervical posture.

Hence, there is an urgent need to bring visibility to wearables that incorporate corrective feedback, as they can aware changes in the daily work routines and, consequently, in the quality of life and work of healthcare professionals. This could result in fewer injuries, lower absenteeism rates, reduced healthcare costs, and increased productivity [79].

In addition to healthcare professionals, there are other fields, particularly in the construction industry, who have a high prevalence of WRMSDs due to daily exposure to excessive effort, sustained and incorrect postures, handling and carrying heavy loads, repetitive tasks, and vibration from work tools [13,14,81]. Based on scientific evidence, it is widely recognised that the construction industry is one of the most dangerous and physically demanding occupations in terms of ergonomics, with a high rate of early retirement and a significant proportion of ageing workers, which increases susceptibility to WRMSDs [20,85]. Choi et al. [58] also support these statements, emphasising that this sector is a promising area for the application of wearables in real-world settings, highlighting the need for research.

With regard to the other included studies, warehouse that involve manual handling of loads is another task that evidences more investigation. It is also worth noting that the study by Lind et al. [52]was carried out in a specific area within the company, which may have minimised the conditions of stress, fatigue, and inherent noise in the workplace, potentially biasing the results as certain variables were under control, thus limiting the generalisability of the findings.

### 4.4. Feedback Source and Type

Wearables have emerged as a solution for the prevention of WRMSDs due to their versatility, particularly because of the real-time feedback they can provide [59]. This feedback should provide personalised information, and it is important to ensure that workers understand and adjust their posture based on the stimulus received [86]. Currently, there are different types of sensory feedback, including haptic, visual, and auditory feedback [9], with haptic feedback being considered the most suitable and common approach [9,31,32,72]. This is consistent with the results of the present study, in which half of the authors used haptic feedback by generating vibratory stimuli. On the other hand, Lee et al. [7] found different results, with sound being the preferred variable. However, considering that sound feedback has a greater potential to distract the worker, the choice of haptic feedback seems to be more appropriate [72]. Indeed, this modality has the added advantage of being intuitive and safe, allowing the worker to maintain concentration on the task at hand, as it only acts in a specific area [31].

On the other hand, Wang et al. [32] recommended the combined use of multiple types of feedback to minimise potential disadvantages and fill gaps. This recommendation is in line with the study by Bootsman et al. [51], which used all three types of sensory feedback. Although this approach has its advantages, it is important to consider the suitability of the region and context for monitoring and applying feedback.

Given that both auditory and haptic feedback do not require visual attention during the task [32], they appear to be a valuable combination. However, in this specific case, the auditory feedback may have been drowned out by the noise of other equipment in the neonatal intensive care unit, which is typically loud. It is therefore important to carefully assess the pertinence of the feedback used, taking into account the aforementioned factors [20].

Doss et al. [49] and Ribeiro et al. [43,53] also used auditory feedback in healthcare professionals, but considering that the transmitted sound can be annoying both for the worker and for patients, it may have a negative impact and act as an inhibiting factor for concentration when the worker is performing a complex task [9]. On the other hand, these constraints resulting from auditory feedback can compromise its effectiveness due to hearing difficulties or competition with other sounds present, especially in environments with high noise levels [72], such as healthcare units or industrial facilities.

Consequently, the use of auditory feedback in the study by Yan et al. [48] may not have been the most suitable choice, as it involved construction tasks where, in addition to the existing noise, some hazardous tasks require full concentration. This may be the reason why the studies by Lind et al. [52,54] opted for haptic feedback for industrial workers.

Visual feedback was also identified in this review. It allows the worker to visualise and correct their movements/posture, and this stimulus is widely used for correcting upper trunk posture [71].

Hence, it seems to be fundamental to have a consensus regarding the timing, quantity, and distance between feedback sensors to minimise accommodation and enhance its effectiveness and usability.

Regarding the application of feedback, an approach that has been widely used is through the sensor itself [31]. Additionally, wearables accompanied by smartphone applications have emerged as a possibility for providing feedback to raise workers’ awareness of their postural behaviours and induce correction [87]. This source of feedback was found in four studies of this review, with the type of feedback provided by the smartphone varying between visual, auditory, or haptic, which is consistent with the study by Wang et al. [88]. Thus, the choice of the source depending on the specific application objective, context, and the better worker acceptance. It is important to note that the smartphone application seems to be easier and more accessible. Therefore, the choice between the sensor or smartphone should always be based on specific needs and worker preferences.

### 4.5. Results after Feedback Application

The included studies demonstrated that there was generally an improvement in posture after the application of haptic feedback in real-world settings, which is congruent with the studies by Kuo et al. [89] and Lind et al. [78], although these were conducted in laboratory settings. The studies by Ribeiro et al. [43], Yan et al. [48], and Doss et al. [49] reported postural improvements following auditory feedback, which is supported by the laboratory study by Boocock et al. [90]. On the other hand, Ribeiro et al. [53] concluded that this type of feedback did not promote improvement during work, suggesting that haptic feedback may have been more appropriate for this class of professionals.

The terminal visual feedback identified in the studies by Thanathornwong et al. [45] and Thanathornwong et al. [44] also demonstrated significant postural improvements, although the stimulus was presented to the worker only at the end of the task. Based on these findings, it can be inferred that the choice of feedback depends on the tasks and the work context being evaluated.

### 4.6. Limitations of the Study

In the time frame defined for this review, it is believed that the COVID-19 pandemic may have contributed to few studies on this topic since 2020. Heterogeneity of the included studies is also a limitation.

### 4.7. Suggestions for Future Studies

Although several studies have identified the non-dominant upper limb, in addition to the cervical region, as having the highest ergonomic risk, and considering that it is associated with a higher risk of developing musculoskeletal disorders, it may be important in future studies to try to incorporate feedback sensors to correct the posture of different segments of the upper limb (especially the wrist, as it is one of the most affected anatomical regions). Furthermore, future research should focus on the integration of textile wearables, improving usability, and wearability as well as clinical validation. The latter is considered to be very important in leading the way for implementation in clinical practice. Future studies should explore wearable solutions with more flexible materials that adapt to the body, with greater power supply optimisation, with the aim of improving the ease and precision of data collection and improving practicality and portability, respectively. Further research should analyse the effectiveness of different types of feedback in work contexts, assessing how these approaches can influence postural improvement, thus guiding the development of personalised interventions.

The included studies in this review do not complete the knowledge storytelling, and gaps that remain in the literature were identified. Currently, research into wearables for postural monitoring and feedback in the workplace is predominantly in an exploratory stage, with a variety of occupations evaluated but with significant emphasis on healthcare professionals and industrial workers. Thus, greater diversification in the occupations study could improve the applicability of wearables in a variety of work contexts.

Most of the integrated studies used prototypes, and there is still a need for more comprehensive clinical validation to guarantee the effectiveness and reliability of wearables for postural monitoring and feedback in a work context.

The duration of postural improvements after discontinuing use of the wearable is still not fully understood, as it is the relative influence of different types of feedback that promote effective postural changes.

Therefore, in order to fill these gaps, longitudinal studies to assess the durability of postural changes after continuous use and after discontinuation of the wearable may be pertinent. On the other hand, it will be important to carry out robust clinical trials to validate the effectiveness of postural monitoring and feedback wearables in the workplace. It will also be pertinent to explore methods for personalising the type of feedback based on individual characteristics and occupational needs.

In addition, it would be relevant to carry out studies with the aim of monitoring through magnetic resonance imaging, the recommended time of use of monitoring wearables and postural feedback to promote postural changes that reduce the impact of WRMSDs. It is also crucial to investigate the durability of postural changes after discontinuing the use of the wearable, contributing to a more comprehensive understanding of the long-term effects.

## 5. Conclusions

The studies predominantly relied on prototype wearables, mainly based on triaxial—but also on six and nonaxial—IMUs, with a focus on kinematic variables of human movement.

The spine stands out as a location for sensor placement, in varying numbers, to monitor and provide feedback on specific tasks performed by healthcare professionals, as well as those in the industry and construction sectors. Personal protective equipment and uniforms were the preferred location for sensor attachment.

Visual, auditory, and haptic feedback were identified in this review, with particular emphasis on the latter for improving posture during work activities and with the most common source being the sensor itself.

## Figures and Tables

**Table 1:** Eligibility criteria according to PCC.

Criteria	
Population	Active adults assessed in the context of work tasks
Concept	Use of wearables to monitor and correct work-related postural changes through sensory biofeedback
Context	Workstation

**Table 2:** Search Strategies in different databases.

Database	Search Strategies
PubMed^®^	(posture OR “postural assessment” OR “body posture” OR “Postural Analysis” OR “posture monitoring” OR “postural correction”) AND (“Wearable Devices” OR Wearables OR “wearable systems” OR “commercial wearable” OR textiles OR sensor* OR “inertial sensor” OR “sensor system” OR “sensor network” OR “smart sensor” OR “pressure sensor” OR “plantar sensor” OR IMU OR gyroscope OR magnetometer OR electromyography OR *feedback) AND (Workplace OR “work-related musculoskeletal disorder” OR “real-time measurement” OR Industry OR “work-station” OR “real-context”) NOT (stress OR exoskeleton OR “Physical activity” OR Physiological)
Web of Science^®^ (WOS)	AK = ((wearable OR postural wearable OR commercial wearable OR textiles OR sensor*) AND (workplace OR workstation OR office WORK OR work-related musculoskeletal disorder) AND postur*) OR AB = ((wearable OR postural wearable OR commercial wearable OR textiles OR sensor*) AND (workplace OR workstation OR office WORK OR work-related musculoskeletal disorder) AND postur*) OR AB = (Wearable AND sensor* AND workplace) OR TI = (Wearable AND sensor* AND workplace) AB= ((posture OR “postural assessment” OR “body posture” OR “Postural Analysis” OR “posture monitoring” OR “postural correction”) AND (‘Wearable Electronic Devices’ OR ‘Wearables’ OR “wearable systems” OR “postural wearable” OR “ commercial wearable” OR textiles OR sensors OR sensor OR “inertial sensor” OR “ sensor system” OR “sensor network” OR “smart sensor” OR “pressure sensor” OR “plantar sensor” OR IMU OR gyroscope OR magnetometer OR electromyography OR feedback) AND (Workplace OR “work-related musculoskeletal disorder” OR “real-time measurement” OR Industry OR “work-station” OR “real- context”))
Scopus^®^	Posture AND Wearable AND Workplace
Google Scholar^®^	(“Postural Analysis” OR “Postural Correction”) AND (Wearable* OR *feedback) AND Workplace

## Data Availability

Not applicable.

## Supplementary Materials

The following supporting information can be downloaded at: https://osf.io/z93ah (accessed on 3 June 2023), the protocol for this scoping review.
